# RuBisCO in Non-Photosynthetic Alga *Euglena longa*: Divergent Features, Transcriptomic Analysis and Regulation of Complex Formation

**DOI:** 10.1371/journal.pone.0158790

**Published:** 2016-07-08

**Authors:** Kristína Záhonová, Zoltán Füssy, Miroslav Oborník, Marek Eliáš, Vyacheslav Yurchenko

**Affiliations:** 1 Life Science Research Centre, Department of Biology and Ecology and Institute of Environmental Technologies, Faculty of Science, University of Ostrava, 701 00 Ostrava, Czech Republic; 2 Institute of Parasitology, Biology Centre ASCR, 370 05 České Budějovice, Czech Republic; 3 University of South Bohemia, Faculty of Science, 370 05 České Budějovice, Czech Republic; 4 Institute of Microbiology ASCR, Centrum Agaltech, 379 01 Třeboň, Czech Republic; University of Freiburg, GERMANY

## Abstract

*Euglena longa*, a close relative of the photosynthetic model alga *Euglena gracilis*, possesses an enigmatic non-photosynthetic plastid. Its genome has retained a gene for the large subunit of the enzyme RuBisCO (*rbcL*). Here we provide new data illuminating the putative role of RuBisCO in *E*. *longa*. We demonstrated that the *E*. *longa* RBCL protein sequence is extremely divergent compared to its homologs from the photosynthetic relatives, suggesting a possible functional shift upon the loss of photosynthesis. Similarly to *E*. *gracilis*, *E*. *longa* harbors a nuclear gene encoding the small subunit of RuBisCO (RBCS) as a precursor polyprotein comprising multiple RBCS repeats, but one of them is highly divergent. Both RBCL and the RBCS proteins are synthesized in *E*. *longa*, but their abundance is very low compared to *E*. *gracilis*. No RBCS monomers could be detected in *E*. *longa*, suggesting that processing of the precursor polyprotein is inefficient in this species. The abundance of RBCS is regulated post-transcriptionally. Indeed, blocking the cytoplasmic translation by cycloheximide has no immediate effect on the RBCS stability in photosynthetically grown *E*. *gracilis*, but in *E*. *longa*, the protein is rapidly degraded. Altogether, our results revealed signatures of evolutionary degradation (becoming defunct) of RuBisCO in *E*. *longa* and suggest that its biological role in this species may be rather unorthodox, if any.

## Introduction

The plastid is a semi-autonomous organelle. Its functionality depends on coordinated expression of nuclear and plastid genes. One of the best known examples of such coordination is ribulose-1,5-bisphosphate carboxylase/oxygenase (RuBisCO), an enzyme catalyzing the very first reaction of the Calvin-Benson cycle, in which CO_2_ is incorporated into organic matter [1]. RuBisCO also catalyzes a competing reaction, oxygenation of ribulose-1,5-bisphosphate in the photorespiration pathway [2]. The plastid RuBisCO holoenzyme is composed of octamers of two different subunits. The only known exceptions were documented in dinoflagellates and chromerid algae, where RuBisCO comprises just one nuclear genome-encoded subunit (form II RuBisCO). In these lineages, the *Rbc* gene was acquired from a bacterium by horizontal gene transfer [3]. In all other cases, the large subunit, possessing the catalytic activity of the holoenzyme, is always encoded in the plastid genome by the *rbcL* gene and synthesized on the plastid ribosomes in the stroma [4,5]. The *rbcS* gene, encoding the small non-catalytic RuBisCO subunit, is also located in the plastid genome in glaucophytes, rhodophytes and organisms with rhodophyte-derived secondary plastids. However, in green algae and land plants (Chloroplastida), and organisms with green algal-derived plastids, the small subunit is encoded in the nucleus, synthesized on the cytoplasmic ribosomes and post-translationally imported into the plastid [6,7]. The biological functions of the small subunit are not well understood. It plays a structural role by stabilizing the mature holoenzyme and is required for maximal catalytic activity and specificity of the large subunit [4,8]. The small subunit may also be responsible for assembling RuBisCO in pyrenoids and may serve as a CO_2_ reservoir [5,9].

The genus *Euglena* is the eponymous taxon of euglenophytes, an algal group nested in the phylum Euglenozoa comprising mostly plastid-less organisms such as kinetoplastids, diplonemids, and primarily aplastidic euglenids [10]. It is now established that euglenophytes have evolved from a phagotrophic euglenid ancestor by acquisition of a plastid through engulfment of a green alga related to the extant prasinophyte genus *Pyramimonas* via secondary endosymbiosis [11,12]. Most euglenophytes, including the best studied species *Euglena gracilis*, harbor photosynthetically active plastids, but can also grow heterotrophically (osmotrophically [13]). Interestingly, several euglenophyte lineages independently resorted to the exclusively heterotrophic nutritional mode by losing photosynthesis (e.g. *Cyclidiopsis acus*, *Euglena hyalina*). The fate of the plastid in most of these species has not been investigated [14]. However, at least one of the secondarily non-photosynthetic euglenophytes, *Euglena longa* (previously known as *Astasia longa*), apparently harbors a cryptic plastid, as evidenced from a complete plastid genome sequence [15].

*Euglena longa* is a close relative of *E*. *gracilis* [14], hence this species pair provides a unique opportunity for investigations of evolution and function of non-photosynthetic plastids. The difference between the two species is reflected in the size of their plastid genomes–the one from *E*. *longa* comprises 73.345 kb, which is half the size of the genome of the photosynthetic plastid from *E*. *gracilis* (143.170 kb) [15,16]. The sets of genes encoding proteins involved in transcription and translation are nearly identical, except for the *rps18* gene missing from *E*. *longa*. In both species the genome harbors a region comprising three tandemly arrayed operons, each including the 16S, 23S, and 5S rDNA genes, as well as one additional adjacent stand-alone copy of the 16S rDNA gene [15,16]. However, all the genes encoding photosynthesis-related proteins are absent from the plastid genome of *E*. *longa*, with the salient exception of the *rbcL* gene [15,17]. This gene contains seven group II introns, whereas its ortholog in *E*. *gracilis* possesses nine introns. The amino acid identity of the RBCL proteins in these two species is 82% and the expression of the *rbcL* gene in *E*. *longa* was confirmed by northern and western blotting analyses [17].

As in other eukaryotes with a “green” plastid, all euglenophyte plastid genomes sequenced to date lack an *rbcS* [18], so a nuclear version (*RbcS*, using a standard notation for nuclear genes) is expected to be present in these species. This has primarily been investigated in *E*. *gracilis*, where the nuclear genome-encoded small subunit of RuBisCO (RBCS) is synthesized as a polyprotein with a molecular weight of approximately 130 kDa [19]. Very recently, partial *RbcS* cDNA sequences from various euglenophytes were reported, but without any detailed analysis [20].

The *E*. *gracilis* RBCS polyprotein includes an array of eight small subunits separated by linker decapeptides. The N-terminal region of the nascent polyprotein represents a tri-partite targeting sequence [19,21]. It starts with a signal peptide, which directs the pre-protein to the endoplasmic reticulum, where it is presumably cleaved off. The second part is a stop-transfer sequence, which is believed to anchor the protein in the membrane of a transport vesicle *en route* to the plastid. The third part is represented by the transit peptide mediating the import of the polyprotein into the plastid stroma, and is found at the N-terminus of the first subunit [21]. Upon translocation to the stroma, the RBCS polyprotein is finally processed by removing the transit peptide and by excision of the linker decapeptides [19,22]. Mature RuBisCO small subunits of *E*. *gracilis* have a molecular weight of approximately 15 kDa and together with large subunits compose the RuBisCO holoenzyme [23].

The presence of an apparently functional *rbcL* gene in the *E*. *longa* plastid genome raises a question about its actual biological role in the absence of a photosynthetic apparatus in this species. This is the only protein-coding gene in the *E*. *longa* plastid genome with a function not directly related to gene expression. Thus, this gene might be the *raison d'etre* for maintaining the plastid genome by *E*. *longa*. Importantly, the *rbcL* gene has been kept by some other non-photosynthetic plastids (for example in those species from the plant parasitic family Orobanchaceae) [24–26]. As a step towards understanding the biological significance of the plastid RuBisCO in *E*. *longa* (and in non-photosynthetic eukaryotes in general), here we present new data on its expression and individual subunits' stability.

## Materials and Methods

### Culture conditions, RNA isolation and cDNA synthesis

*Euglena longa* strain CCAP 1204-17a and *E*. *gracilis* strain Z (hereafter denoted as heterotrophic EL and mixotrophic EG+, respectively) were cultivated statically under constant illumination at 23°C in Cramer-Myers medium [27] supplemented with ethanol (0.8% v/v). *E*. *gracilis* strain Z was also cultivated photosynthetically, i.e. without addition of ethanol or any other source of organic carbon (hereafter denoted as EG-). The cultures of *E*. *longa* were not completely axenic, but the contaminating bacteria were kept at as low level as possible. RNA was isolated using RNeasy Plus Universal Mini Kit (Qiagen, Hilden, Germany). cDNA synthesis was carried out with random hexanucleotide primers using Transcriptor First Strand cDNA Synthesis Kit (Roche, Basel, Switzerland).

### PCR and quantitative reverse-transcription PCR

Sequences of all primers used are listed in S1 Table. The *RbcS* was amplified from 10 ng of *E*. *longa* cDNA using primers RbcS_F1 and RbcS_R and Herculase II Fusion DNA Polymerase (Agilent Technologies, Santa Clara, USA), and PCR conditions as follows: 95°C for 1 min; 30 cycles of 95°C for 20 sec, 65°C for 20 sec, 68°C for 5 min, and the final extension at 68°C for 4 min. The most abundant band was purified from the gel and sequenced directly.

Quantitative RT-PCR experiments were performed according to the manufacturer’s instructions using LightCycler® 480 SYBRGreen Master mix (Roche) as described earlier [28]. All measurements were done in triplicates. Standard curve method for relative quantification and expression of the 18S ribosomal RNA gene was used for normalization [29].

### Protein blotting, half-life determination and mass spectrometry

Cell cultures were treated with 20 μg/ml of cycloheximide (Sigma-Aldrich, St. Louis, USA) as described elsewhere [30] and aliquots were taken at 0, 1, 4, 8, and 24 h post treatment. The *Euglena* spp. cells were lysed in RIPA buffer (Thermo Scientific, Waltham, USA) with protease inhibitor cocktail (Roche) as described previously [31].

Twenty-five μg of total protein were separated by SDS-PAGE and subjected to western blotting and immunodetection using polyclonal rabbit anti-RBCS (AS07 222, 1:1,000), polyclonal chicken anti-RBCL (AS01 017, 1:10,000), and polyclonal rabbit anti-Tubulin (AS10 680, 1:2,000) antibodies. All primary antibodies were from Agrisera, Vännäs, Sweden. For detection, the HRP-labeled goat anti-chicken IgY H&L (1:10,000, ab97135, Abcam, Cambridge, UK) and donkey anti-rabbit (1:5,000, RPN2108, GE Healthcare Bio-Sciences, Pittsburgh, USA) secondary antibodies were used. The membranes were treated with ECL™ Western Blotting Analysis System (GE Healthcare Bio-Sciences). For RBCL detection in the case of *E*. *longa*, the SuperSignal West Femto Maximum Sensitivity Substrate kit (Thermo Scientific) was used.

Identity of the *E*. *longa* large subunit of RuBisCO was confirmed by mass spectrometry. A band was dissected from a polyacrylamide gel in the region corresponding to the expected size of the *E*. *longa* RBCL protein and eluted proteins were analyzed by mass spectrometry using MALDI-TOF/TOF mass spectrometer TOF Impact II (Bruker Co, Billerica, USA) at the Proteomics Core Facility (Central European Institute of Technology, CEITEC, Brno, Czech Republic).

### Sequence searches and phylogenetic analyses

Homologs of the investigated proteins (RBCL, RBCS, RuBisCO activase RCA, and RuBisCO assembly chaperone RAF) were identified by BLAST [32] in the non-redundant protein database at NCBI, in transcriptome assemblies of relevant species generated by the Marine Microbial Eukaryote Transcriptome Sequencing Project (http://marinemicroeukaryotes.org/) [33], and in our unpublished transcriptome of *E*. *longa* obtained by assembling RNA-seq Illumina reads from two differently treated cultures (details on the RNA preparation, RNA-seq, and transcriptome assembly will be published elsewhere). The cDNA sequences corresponding to RCA and RBCS proteins from *E*. *longa* were deposited at GenBank with accession numbers KT818573-KT818576. Accession numbers of all sequences of RBCL, RBCS, and RCA proteins analyzed in this study are listed in S2, S3, and S4 Tables, respectively. Sequences were aligned using MAFFT 7 (Multiple Alignment using Fast Fourier Transform) [34]. The alignment was manually refined using BioEdit 7.1.7 and ambiguously aligned positions were removed [35]. The resulting RBCL alignment contained 53 sequences and 473 amino acid positions, the RBCS alignment contained 42 sequences and 122 amino acid positions, and the RCA alignment contained 30 sequences and 301 amino acid positions. Maximum likelihood (ML) trees were inferred from the alignments using RAxML 8.1.11 employing the strategy of rapid bootstrapping followed by a "thorough" ML search on the original dataset with the LG+Γ substitution model (1,000 bootstrap replicates) [36]. In addition, a Bayesian phylogeny was inferred using PhyloBayes 3.3b with the following parameters: 15,000 generations under the C20 model with Poisson exchange rate, sampling every 100 generations, and maximum divergence allowed set to 0.1 [37].

## Results

### The RBCL sequence of *E*. *longa* is extremely divergent

**Fig 1:**
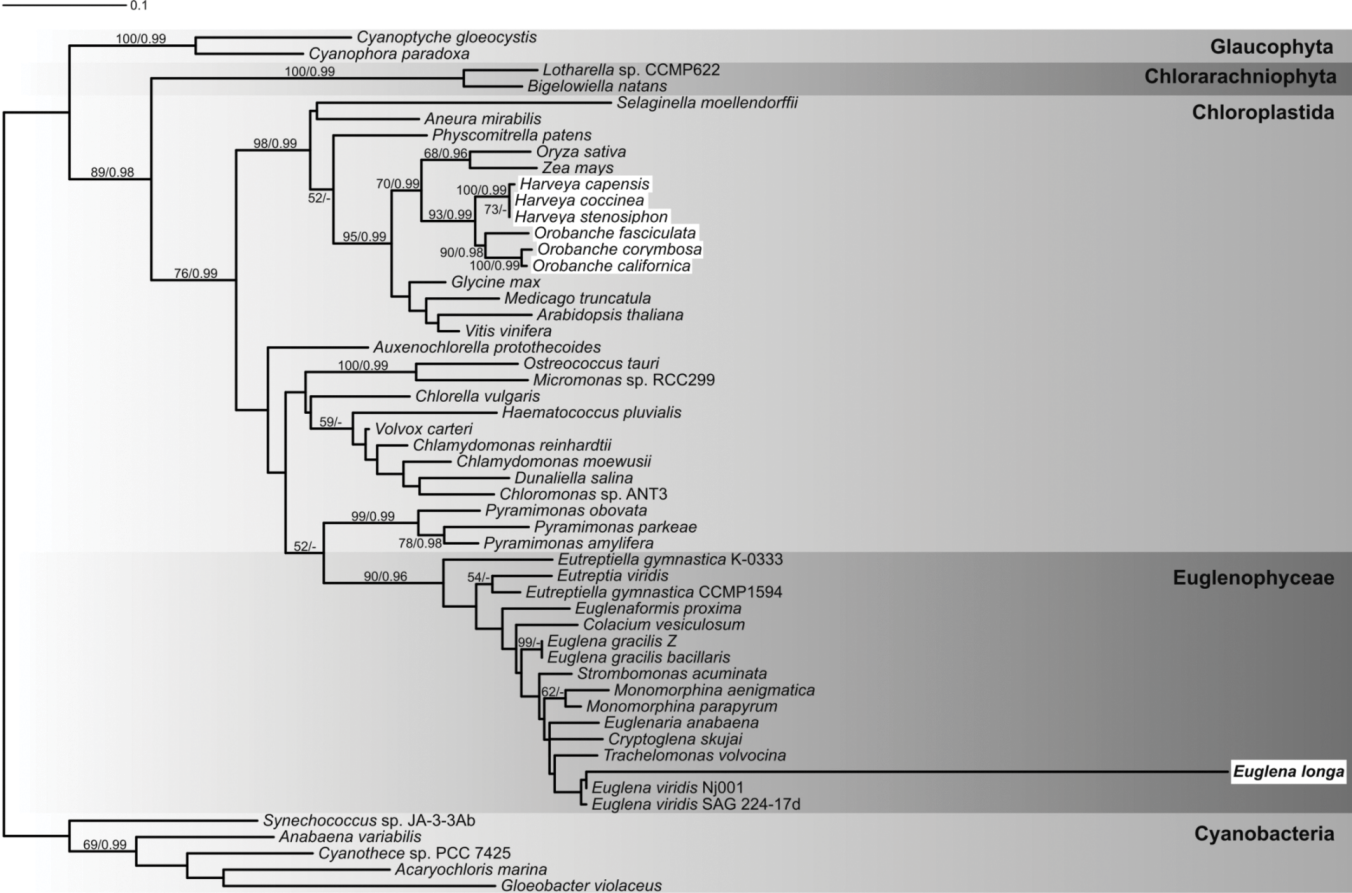
Phylogenetic tree of RBCL protein sequences. The maximum-likelihood tree was inferred with RAxML using the LG+Γ substitution model. The bootstrap support values and posterior probabilities (from PhyloBayes) are indicated at branches when higher than 50% and 0.95, respectively. Highlighted in white boxes are non-photosynthetic species. *E*. *longa* is in bold.

A recent paper reported phylogenetic analyses of Calvin-Benson cycle enzymes in euglenophytes, but the RBCL protein was not analyzed in that study [20]. Therefore, as a starting point for our analysis, we inferred a phylogenetic tree of a set of RBCL proteins including the sequence from *E*. *longa*. The assembled dataset contained available RBCL sequences from euglenophytes and a selection of sequences from plants and algae possessing the cyanobacteria-derived form of RuBisCO. It excluded distantly related RBCL sequences from rhodophytes and algae with rhodophyte-derived secondary plastids comprising a proteobacteria-derived RuBisCO form [38]. The phylogenetic analysis confirmed that euglenophyte RBCL sequences are monophyletic and constitute a sister lineage to sequences from *Pyramimonas* spp. (Fig 1). Although the Euglenophyceae-*Pyramimonas* clade has low statistical support, it is consistent with current views on the origin of the euglenophyte plastid. The *E*. *longa* RBCL is nested among euglenophyte homologs in the tree. Remarkably, its branch is extremely long, reflecting a high number of substitutions in the sequence compared to other euglenophytes analyzed, all of which were photosynthetic. All amino acid involved in the protein assembly or responsible for its carboxylation/oxygenation catalytic function—T65, S112, N123, K128, K177, L290, R295, G322, H327, V331, K334, L335, A378, S379, G381, and G404 [2,39]—were conserved in *E*. *longa*.

### RBCS in *E*. *longa* is encoded as a precursor polyprotein including eight RBCS repeats, one of which is highly divergent

Given the extreme divergence of the RBCL sequence in *E*. *longa*, we searched our deeply sequenced transcriptome of this species for the presence and possible unusual features of homologs of selected interacting partners of RBCL. The first obvious candidate was RBCS, and we indeed found three contigs corresponding to the *RbcS* gene. The first contig included a predicted N-terminal plastid-targeting sequence and a single domain corresponding to the mature RBCS, highly similar to the RBCS sequence of *E*. *gracilis*. The second contig contained the N-terminally truncated (without the plastid-targeting sequence) RBCS-like region followed by a linker decapeptide highly similar to that separating RBCS repeats in the precursor polyprotein in *E*. *gracilis*. The third, short contig translated into a truncated protein that included the C-terminal half of the decapeptide linker and a region resembling the N-terminal part of the RBCS monomer.

We reasoned that similarly to *E*. *gracilis* [19], *E*. *longa* may also encode a precursor RBCS polyprotein with repeated RBCS units separated by linker decapeptides, but that the actual cDNA sequence was not properly assembled from the NGS data due to its repeated nature. In addition, the presence of two apparently truncated contigs suggested that the polyprotein may include two types of RBCS-like sequences: one highly similar to RBCS of *E*. *gracilis* and one rather different from it. To test this, we prepared cDNA from *E*. *longa* and performed PCR with a forward primer matching the 5’-end of the presumed complete coding sequence of the *RbcS* mRNA (i.e. the region coding for the first several amino acids of the signal peptide) and a reverse primer matching the presumed 3’-UTR of the *RbcS* mRNA (RbcS_F1 and RbcS_R, respectively, S1 Table and boxed in black in S1A Fig). The reaction yielded eight products of different length, with the longest one over 4 kb and the shortest around 800 bp. Importantly, the seven shorter products differed in length by about 420 bp (a size of a single repeat of the RBCS monomer plus a decapeptide linker). We interpret this result as evidence for the existence of a long repeated *RbcS* mRNA molecule (S1B Fig) similar in size to that of *E*. *gracilis* [19]. Furthermore, we assume that the mRNA includes eight RBCS repeats and the shorter PCR products originated from illegitimate pairing of incompletely amplified DNA strands with the repeated sequence.

We sequenced the ends of the most abundant PCR product with the primers used for PCR. Sequencing using the RbcS_F1 primer yielded the complete N-terminal plastid-targeting sequence connected with the first *RbcS* unit. The reverse primer (RbcS_R) returned a partial sequence of the last unit adjacent to the 3'-UTR. Using an internal primer matching the mRNA sequence just upstream of the first presumed RBCS repeat (RbcS_F2, S1 Table and boxed in dark blue in S1A Fig) produced a sequence comprising two complete and one 3'-truncated RBCS repeats separated by two linker regions.

The third incomplete repeat contained three synonymous substitutions as compared to the first two repeats, indicating some degree of variation in the repeat sequences (S1A Fig). The predicted linker decapeptide (NMAAMTGEKD) differed from the *E*. *gracilis* linker sequence in only one amino acid (Asn instead of Gly at the first position). These results indicated that the contig in the transcriptome assembly with an ORF encoding the plastid-targeting sequence followed by only one RBCS region is an assembly artifact. In fact, *E*. *longa* shares a similar repeated structure of the RBCS precursor polyprotein with *E*. *gracilis*.

We then designed a primer matching a region close to the 5'-end of the coding sequence of the highly divergent RBCS-like unit (RbcS-X_F in S1 Table and boxed in dark blue in S1A Fig). The resulting sequence confirmed the existence of a single continuous sequence encoding a highly divergent variant of the RBCS unit. It is characterized by multiple amino acid changes and three in-frame deletions (one of one amino acid, and two of five amino acids). This divergent RBCS sequence is followed by a linker decapeptide (a variant with the first Asn residue substituted by Ser) and a full canonical RBCS repeat (S1A and S1C Fig).

A phylogenetic analysis of RBCS sequences (including the canonical but not the divergent RBCS from *E*. *longa*) demonstrated monophyly of Euglenophyceae sequences (S2 Fig). The euglenophyte RBCS clade expectedly formed a lineage within a group of sequences from the Chloroplastida, but its precise position could not be determined, perhaps because of the very short length of the RBCS sequences. In contrast to RBCL, the (canonical) *E*. *longa* RBCS sequence does not seem to be divergent compared to sequences from other euglenophytes, so no apparent change in the functional mode of this protein is evident from this analysis.

### Expression of RuBisCO small and large subunits is severely repressed in *E*. *longa*

**Fig 2:**
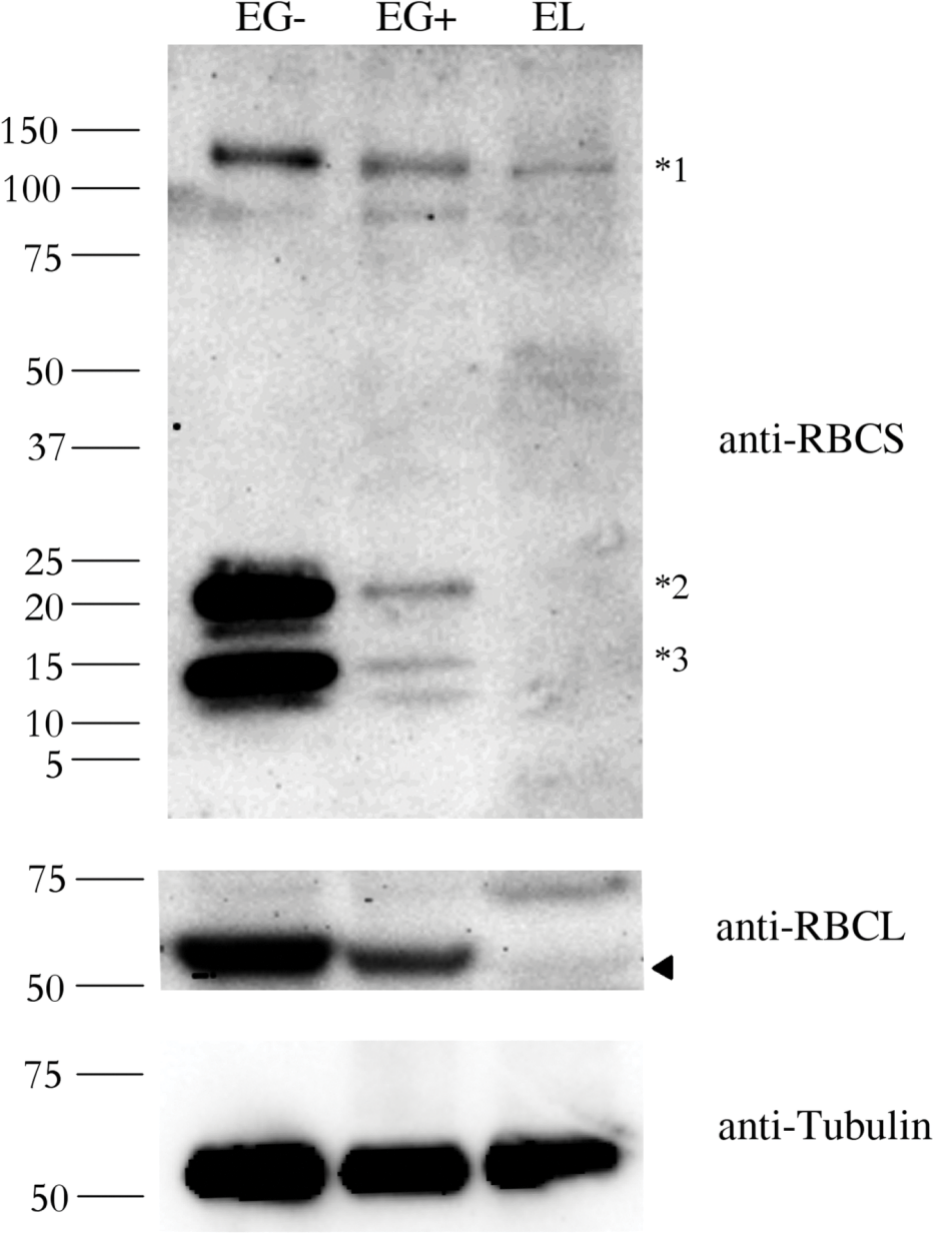
Abundance of the RBCS and RBCL proteins in *Euglena gracilis* and *Euglena longa*. Protein immunodetection was performed using anti-RBCS, anti-RBCL, and anti-Tubulin antibodies. Three bands with different molecular weights were observed in anti-RBCS immunoblotting. The ~130 kDa band (marked *1) corresponds to polyprotein synthesized in the nucleus. The ~15 kDa band (marked *3) corresponds to the processed monomer after cleavage of the signal sequence and excision of decapeptides. The ~22 kDa band (marked *2) possibly corresponds to a monomer still attached to the transit peptide. The identity of the RBCL protein (arrowhead in the anti-RBCL panel) was confirmed by mass-spectrometry. Tubulin served as a loading control. Molecular weights in kDa are indicated on the left. EG-, *E*. *gracilis* cultivated photosynthetically (without ethanol); EG+, *E*. *gracilis* cultivated mixotrophically (with ethanol); EL, *E*. *longa*.

To further explore differences between the RuBisCO enzyme in the photosynthetic *E*. *gracilis* and the non-photosynthetic *E*. *longa*, we analyzed RBCS and RBCL protein levels in both species. As documented before [19,40], we observed three bands with different molecular weight using an anti-RBCS antibody in photosynthetically grown *E*. *gracilis* (EG-) (Fig 2, anti-RBCS panel). The band with a molecular weight of ~130 kDa (marked *1 in Fig 2) corresponds to the polyprotein synthesized in the cytosol. The smallest band (~15 kDa; marked *3 in Fig 2) corresponds to the processed monomers after cleavage of the signal sequence and excision of decapeptides. The labeled peptides with a molecular weight of ~22 kDa (marked *2 in Fig 2) presumably corresponds to monomers still attached to the transit peptide [40]. All these peptides were also observed in mixotrophically grown *E*. *gracilis* (EG+), but *RbcS* expression has significantly decreased. This difference may correlate with a change from the autotrophic (EG-) to the mixotrophic (EG+) conditions. It is expected that the presence of another carbon source (ethanol) results in the reduced expression of the photosynthesis-related genes.

In *E*. *longa* (EL) extracts, only the polyprotein of ~130 kDa was detected in a substantially lower amount compared to both EG+ and EG- samples. In this case, no monomers were observed in immunoblotting experiments. Absence of small molecular weight RBCS proteins (i.e. ~15 and ~22 kDa) was also confirmed by mass-spectrometry. These results suggest that RBCS polyprotein is not properly processed and/or transported in *E*. *longa* cells.

A similar pattern was observed for the RBCL protein (Fig 2, anti-RBCL panel), i.e. the RBCL protein was most abundant in EG-, its abundance decreased in EG+ and it was barely detectable in EL. The anti-RBCL antibody also recognized a non-specific band (74 kDa) apparent especially in the EL extract. The identity of the less intense band as RBCL in EL (marked by arrowhead in Fig 2, anti-RBCL panel) was confirmed by mass spectrometry.

### Abundance of the RBCL protein correlates with the mRNA level in both *Euglena* species, but abundance of the RBCS protein in *E*. *longa* is determined primarily by its rapid turnover

**Fig 3:**
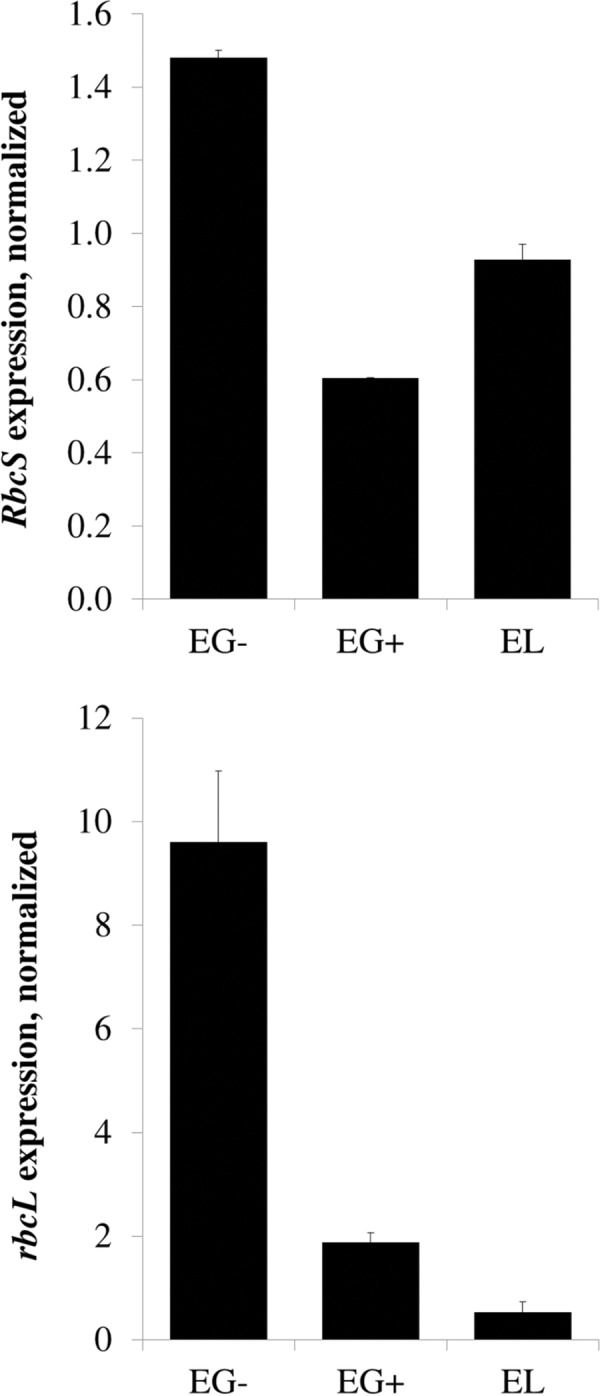
Expression of the *RbcS* and *rbcL* genes in *Euglena gracilis* and *Euglena longa*. Expression levels of *RbcS* and *rbcL* mRNAs were analyzed by quantitative RT-PCR and normalized over the 18S ribosomal RNA. Cultivation conditions and species are denoted as in Fig 2.

In order to investigate the molecular mechanism behind the different RuBisCO subunit abundances in different *Euglena* species or cultivation conditions, we determined *RbcS* and *rbcL* mRNA levels of RuBisCO subunits using quantitative RT-PCR. The *rbcL* transcript level was highest in EG-, substantially lower in EG+, and very low in EL (Fig 3). The level of *rbcL* mRNA correlated well with the protein abundance (compare Fig 2 and Fig 3). This is not the case for the *RbcS* mRNA and RBCS protein. Even though the *RbcS* transcript level was slightly decreased in EG+ and EL compared to EG- (Fig 3), the magnitude of the difference does not correspond to the dramatic difference in the RBCS protein abundance in these species (Fig 2). These results indicate that post-transcriptional regulation is chiefly responsible for the differences in the RBCS abundance in the different conditions/species.

**Fig 4:**
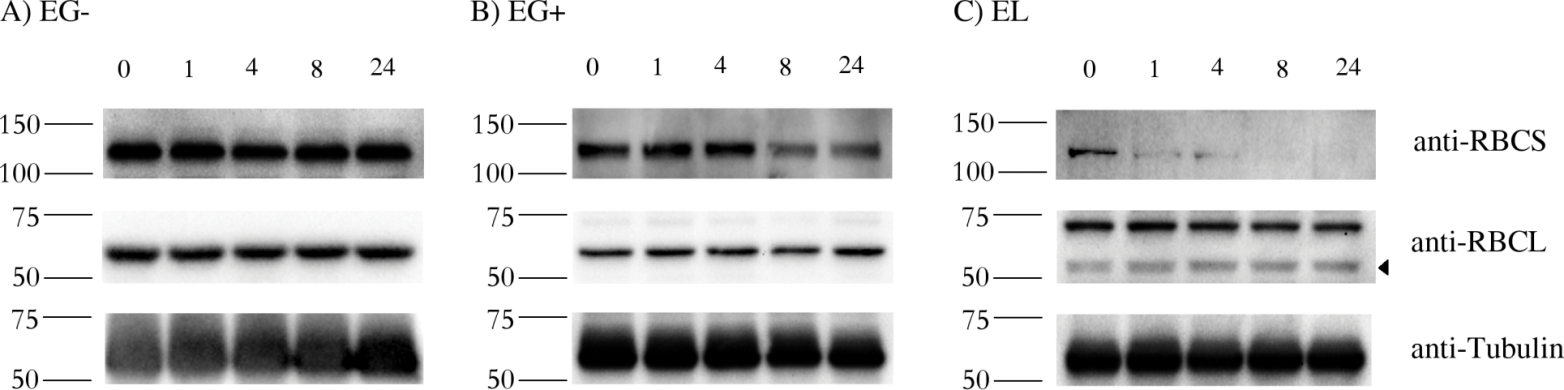
Stability of RBCS and RBCL proteins in *Euglena gracilis* and *Euglena longa*. Cell cultures were treated with 20 μg/ml of cycloheximide, aliquots were taken at 0, 1, 4, 8, and 24 h post treatment, and analyzed by western blotting using anti-RBCS, anti-RBCL and anti-Tubulin antibodies. Molecular weights (in kDa) are indicated on the left of each panel. The identity of the RBCL protein (arrowhead in the anti-RBCL panel) was confirmed by mass-spectrometry. Tubulin served as a loading control. Cultivation conditions and species are denoted as in Fig 2.

One possibility is that the synthesis of the RBCS protein is proportional to the mRNA level, but the protein is less stable in EG+ and EL than in EG-. To test this, *Euglena* cells grown for 10 days were treated with cycloheximide that blocks cytoplasmic translation, and proteins from these cultures were isolated. In EG-, the RBCS precursor polyprotein was stable for at least 24 hours (Fig 4A, anti-RBCS panel). However, it was not as stable in EG+ and was very unstable in EL (Fig 4B and 4C, anti-RBCS panel). The half-life of the polyprotein in *E*. *longa* was estimated to be about 10 minutes.

As expected, the RBCL abundance was not influenced by the cycloheximide treatment (Fig 4, anti-RBCL panel), given that this subunit is encoded by the plastid genome and its synthesis on plastid ribosomes is cycloheximide-insensitive. Tubulin served as a loading control (Fig 4, anti-Tubulin panel) [41].

## Discussion

*Euglena longa* is the closest relative of the photosynthetic euglenophyte alga *Euglena gracilis*. In contrast to its kin, it harbors an enigmatic non-photosynthetic plastid. The only gene for photosynthesis-related protein retained in the *E*. *longa* plastid genome is that encoding the large subunit of the enzyme RuBisCO. It is the first enzyme of the Calvin-Benson cycle and one of the most abundant proteins on Earth.

We showed that the RBCL protein sequence in *E*. *longa* is extremely divergent compared to its homologs from the photosynthetic relatives (Fig 1). This implies a possible functional shift upon the loss of photosynthesis. However, the loss of photosynthesis *per se* does not necessarily cause high divergence observed for the *E*. *longa* sequence. Branches of RBCL sequences coming from *Orobanche* and *Harveya* species, non-photosynthetic angiosperms that have also retained *rbcL* genes in their plastid genomes [25,26], are not extended beyond those coming from their photosynthetic relatives (Fig 1).

*E*. *longa* possesses a nuclear gene encoding the small subunit of RuBisCO. As in its photosynthetic relative *E*. *gracilis*, RBCS is expressed as a precursor polyprotein composed of several repeats. Interestingly, one of those repeats is highly divergent. Our experimental approach could not provide a completely reconstructed sequence of the actual *RbcS* mRNA in *E*. *longa* because of its repeated nature. We could not confirm the actual number of the repeats (both the canonical and the divergent), even though we expect it to be similar to *E*. *gracilis* based on the similarities of their transcripts. We also could not determine the relative position of the divergent and the canonical repeats. At least first three and last two repeats are of the canonical form (S1 Fig).

Both RBCL and the RBCS proteins are expressed in *E*. *longa*, but their abundances are very low compared to *E*. *gracilis*. There are several possible explanations for this observation. 1) Expression of both *RbcS* and *rbcL* genes might be repressed in mixotrophically cultivated *E*. *gracilis* and in *E*. *longa*. 2) Transcription of either *RbcS* or *rbcL* may be inhibited. As demonstrated for other protein complexes [30,42], the RuBisCO complex expression may depend on abundance of its individual subunits. 3) The levels of individual proteins may be regulated post-transcriptionally or post-translationally. In *E*. *gracilis*, post-transcriptional regulation prevails in controlling protein abundance [43].

The processing of the RBCS polyprotein in *E*. *longa* seems to be impaired. We failed to detect monomers of the small subunit even when using more sensitive kit for detection and/or after prolonged membrane exposure. No such defect was observed in *E*. *gracilis* cultivated in the presence or absence of ethanol. In both cases fully and partially processed monomers were readily detectable (Fig 2). Several other proteins, such as phosphoglycerate kinase or numerous subunits of light-harvesting complexes I and II, are translated as polyproteins in *E*. *gracilis* [44–47]. This phenomenon has been scarcely seen in other eukaryote taxa [48]. The polyproteins are transported into the plastid and mature units are released upon cleavage of the interspersing/delineating linkers, typically decapeptides. The linker is supposedly cleaved off by a thiol peptidase unrelated to the signal peptidase or thylakoid processing peptidase [49]. The presence of the linker consensus sequence suggests an involvement of a matrix metallopeptidase-9-like enzyme (as predicted by the Prosper tool [50]). Because of its broad specificity, we assume that the linker sequences in RBCS of *E*. *longa* are the only sites not masked by secondary structures against the proteolytic activity. However, the identity or even the existence of a putative linker peptidase in *E*. *longa* is currently not confirmed. The selective pressure for the presence of the linker peptidase has mostly disappeared with the loss of other photosynthesis-related proteins.

The expression of the RuBisCO proteins in *Euglena* species is regulated post-translationally at the level of the complex formation. Indeed, blocking the cytoplasmic translation by cycloheximide has no immediate effect on the RBCS stability in photosynthetically grown *E*. *gracilis*, but in *E*. *longa*, the protein is rapidly degraded (Fig 4). Stability of the RuBisCO small subunit may depend on the presence or the absence of its binding partner, i.e. octamer of the RBCL protein. Notably, a similar phenomenon has been documented in *Nicotiana tabacum*, where the RBCS protein was undetectable in the absence of the compatible large subunit counterpart [51,52]. The molecular mechanism behind this observation remained unclear. The extremely high turnover rate of RBCS in *E*. *longa* might indicate that its assembly with RBCL is compromised and does not result in the formation of the functional holoenzyme.

RuBisCO subunits interact not only with each other, but also with other protein partners required for the proper assembly and function of the enzyme. One is RuBisCO activase (RCA), an ATP-hydrolyzing enzyme facilitating removal of inhibitory sugars from the RuBisCO holoenzyme [7]. Indeed, our transcriptomic data revealed the presence of a *E*. *longa* RCA homolog (S4 Table). A phylogenetic analysis of the RCA protein sequences confirmed the monophyly of those coming from Euglenophyceae and demonstrated their close relationship with sequences from chlorophytes (S3 Fig). Although the branch of the *E*. *longa* RCA sequence is the longest among euglenophyte sequences analyzed, the difference is by far less dramatic than that exhibited by RBCL sequences (Fig 1). Furthermore, all motifs necessary for proper RCA function [53] are present in *E*. *longa* sequence, suggesting that its actual function may have been retained in *E*. *longa*.

Other interacting partners of RuBisCO are the plastid homologs of GroEL and GroES chaperonins, which fold RBCL monomers into an antiparallel dimer. The RBCL dimers are then assembled into an octamer by the RBCX, bundle sheath defective 2 protein (BSD2), α-carboxysome RuBisCO assembly factor (acRAF) or RAF1/RAF2 proteins [7,54–56]. Binding of RBCS monomers triggers conformational changes of the RBCL octamer and displaces assembly proteins from the complex [7]. The GroEL/GroES homologs possessing predicted plastid-targeting sequences at their N-termini are present in the *E*. *longa* transcriptome (S4 Fig). However, no RBCX, BSD2 or acRAF homologs could be identified in the transcriptomic data from this species. The only protein previously implicated in the RuBisCO assembly identified in the *E*. *longa* transcriptome is RAF. In contrast to higher plants with two homologous RAF1 and RAF2 [55], we found only one homolog of this protein in *E*. *longa*. The biological consequences of this divergence remain to be investigated.

In addition to playing a critical role in photosynthesis, RuBisCO has been implicated in other biochemical processes, such as methionine salvage pathway [57], isomerization of 5-methylthio-D-ribulose-1-phosphate [58], phosphoenolpyruvate carboxylation [59] or sulfur metabolism [60]. It was proposed that in bacteria even the unprocessed RBCS-like polyprotein might function as a scaffold for higher order molecular complexes assemblies [61]. Further functional studies are necessary to delineate RuBisCO functions, if any, in *E*. *longa*.

## Supporting Information

S1 Fig RuBisCO small subunit in *E*. *longa*. (PDF)

S2 Fig Phylogenetic tree of RBCS protein sequences. (PDF)

S3 Fig Phylogenetic tree of RCA protein sequences. (PDF)

S4 Fig GroEL/GroES homologs in *E*. *longa*. (PDF)

S1 Table List of PCR and qPCR primers used in this work. (PDF)

S2 Table List of the RBCL sequences used in phylogenetic analysis. (PDF)

S3 Table List of the RBCS sequences used in phylogenetic analysis. (PDF)

S4 Table List of the RCA sequences used in phylogenetic analysis. (PDF)

## Abbreviations

EG-: photosynthetically grown *E*. *gracilis*
EG+: mixotrophically grown *E*. *gracilis*
EL: heterotrophically grown *E*. *longa*
RCA: RuBisCO activase
RBCL: large subunit of RuBisCO
RBCS: small subunit of RuBisCO
RuBisCO: ribulose-1,5-bisphosphate carboxylase/oxygenase

## References

[1] Bassham JA, Benson AA, Calvin M. The path of carbon in photosynthesis. J Biol Chem. 1950; 185: 781–787. 14774424

[2] Tabita FR, Hanson TE, Li H, Satagopan S, Singh J, Chan S. Function, structure, and evolution of the RuBisCO-like proteins and their RuBisCO homologs. Microbiol Mol Biol Rev. 2007; 71: 576–599. 18063718 10.1128/MMBR.00015-07 PMC2168653

[3] Janouškovec J, Horák A, Oborník M, Lukeš J, Keeling PJ. A common red algal origin of the apicomplexan, dinoflagellate, and heterokont plastids. Proc Natl Acad Sci U S A. 2010; 107: 10949–10954. 10.1073/pnas.1003335107 20534454 PMC2890776

[4] Andrews TJ. Catalysis by cyanobacterial ribulose-bisphosphate carboxylase large subunits in the complete absence of small subunits. J Biol Chem. 1988; 263: 12213–12219. 3137223

[5] van Lun M, Hub JS, van der Spoel D, Andersson I. CO_2_ and O_2_ distribution in RuBisCO suggests the small subunit functions as a CO_2_ reservoir. J Am Chem Soc. 2014; 136: 3165–3171. 10.1021/ja411579b 24495214

[6] Watson GM, Tabita FR. Microbial ribulose 1,5-bisphosphate carboxylase/oxygenase: a molecule for phylogenetic and enzymological investigation. FEMS Microbiol Lett. 1997; 146: 13–22. 8997702 10.1111/j.1574-6968.1997.tb10165.x

[7] Hauser T, Popilka L, Hartl FU, Hayer-Hartl M. Role of auxiliary proteins in RuBisCO biogenesis and function. Nat Plants. 2015; 1: 15065 10.1038/nplants.2015.65 27250005

[8] Genkov T, Spreitzer RJ. Highly conserved small subunit residues influence RuBisCO large subunit catalysis. J Biol Chem. 2009; 284: 30105–30112. 10.1074/jbc.M109.044081 19734149 PMC2781565

[9] Genkov T, Meyer M, Griffiths H, Spreitzer RJ. Functional hybrid RuBisCO enzymes with plant small subunits and algal large subunits: engineered *rbcS* cDNA for expression in *Chlamydomonas*. J Biol Chem. 2010; 285: 19833–19841. 10.1074/jbc.M110.124230 20424165 PMC2888394

[10] Adl SM, Simpson AG, Lane CE, Lukeš J, Bass D, Bowser SS, et al The revised classification of eukaryotes. J Eukaryot Microbiol. 2012; 59: 429–493. 10.1111/j.1550-7408.2012.00644.x 23020233 PMC3483872

[11] Leander BS. Did trypanosomatid parasites have photosynthetic ancestors? Trends Microbiol. 2004; 12: 251–258. 15165602 10.1016/j.tim.2004.04.001

[12] Yamaguchi A, Yubuki N, Leander BS. Morphostasis in a novel eukaryote illuminates the evolutionary transition from phagotrophy to phototrophy: description of *Rapaza viridis* n. gen. et sp. (Euglenozoa, Euglenida). BMC Evol Biol. 2012; 12: 29 10.1186/1471-2148-12-29 22401606 PMC3374381

[13] Busse I, Preisfeld A. Systematics of primary osmotrophic euglenids: a molecular approach to the phylogeny of *Distigma* and *Astasia* (Euglenozoa). Int J Syst Evol Microbiol. 2003; 53: 617–624. 12710635 10.1099/ijs.0.02295-0

[14] Marin B, Palm A, Klingberg M, Melkonian M. Phylogeny and taxonomic revision of plastid-containing euglenophytes based on SSU rDNA sequence comparisons and synapomorphic signatures in the SSU rRNA secondary structure. Protist. 2003; 154: 99–145. 12812373 10.1078/143446103764928521

[15] Gockel G, Hachtel W. Complete gene map of the plastid genome of the nonphotosynthetic euglenoid flagellate *Astasia longa*. Protist. 2000; 151: 347–351. 11212895 10.1078/S1434-4610(04)70033-4

[16] Hallick RB, Hong L, Drager RG, Favreau MR, Monfort A, Orsat B, et al Complete sequence of *Euglena gracilis* chloroplast DNA. Nucleic Acids Res. 1993; 21: 3537–3544. 8346031 10.1093/nar/21.15.3537 PMC331456

[17] Siemeister G, Hachtel W. Structure and expression of a gene encoding the large subunit of ribulose-1,5-bisphosphate carboxylase (*rbcL*) in the colourless euglenoid flagellate *Astasia longa*. Plant Mol Biol. 1990; 14: 825–833. 2102860 10.1007/BF00016515

[18] Bennett MS, Triemer RE. Chloroplast genome evolution in the Euglenaceae. J Eukaryot Microbiol. 2015; 62: 773–785. 10.1111/jeu.12235 25976746

[19] Chan RL, Keller M, Canaday J, Weil JH, Imbault P. Eight small subunits of *Euglena* ribulose-1,5-bisphosphate carboxylase/oxygenase are translated from a large mRNA as a polyprotein. EMBO J. 1990; 9: 333–338. 2105882 10.1002/j.1460-2075.1990.tb08115.x PMC551670

[20] Markunas CM, Triemer RE. Evolutionary history of the enzymes involved in the Calvin-Benson cycle in euglenids. J Eukaryot Microbiol. 2015: 10.1111/jeu.12282 26566594

[21] Durnford DG, Gray MW. Analysis of *Euglena gracilis* plastid-targeted proteins reveals different classes of transit sequences. Eukaryot Cell. 2006; 5: 2079–2091. 16998072 10.1128/EC.00222-06 PMC1694827

[22] Sailland A, Amiri I, Freyssinet G. Amino acid sequence of the ribulose-1,5-bisphosphate carboxylase/oxygenase small subunit from *Euglena*. Plant Mol Biol. 1986; 7: 213–218. 10.1007/BF00021333 24302307

[23] Yokota A, Harada A, Kitaoka S. Characterization of ribulose 1,5-bisphosphate carboxylase/oxygenase from *Euglena gracilis* Z. J Biochem. 1989; 105: 400–405. 2499574 10.1093/oxfordjournals.jbchem.a122676

[24] Wolfe AD, dePamphilis CW. The effect of relaxed functional constraints on the photosynthetic gene rbcL in photosynthetic and nonphotosynthetic parasitic plants. Mol Biol Evol. 1998; 15: 1243–1258. 9787431 10.1093/oxfordjournals.molbev.a025853

[25] Manen JF, Habashi C, Jeanmonod D, Park JM, Schneeweiss GM. Phylogeny and intraspecific variability of holoparasitic *Orobanche* (Orobanchaceae) inferred from plastid rbcL sequences. Mol Phylogenet Evol. 2004; 33: 482–500. 15336681 10.1016/j.ympev.2004.06.010

[26] Randle CP, Wolfe AD. The evolution and expression of RbcL in holoparasitic sister-genera *Harveya* and *Hyobanche* (Orobanchaceae). Am J Bot. 2005; 92: 1575–1585. 10.3732/ajb.92.9.1575 21646175

[27] Cramer M, Myers J. Growth and photosynthetic characteristics of *Euglena gracilis*. Archiv Fur Mikrobiologie. 1952; 17: 384–402.

[28] Záhonová K, Hadariová L, Vacula R, Yurchenko V, Eliáš M, Krajčovič J, et al A small portion of plastid transcripts is polyadenylated in the flagellate *Euglena gracilis*. FEBS Lett. 2014; 588: 783–788. 10.1016/j.febslet.2014.01.034 24492004

[29] Wong ML, Medrano JF. Real-time PCR for mRNA quantitation. Biotechniques. 2005; 39: 75–85. 16060372 10.2144/05391RV01

[30] Yurchenko V, Xue Z, Gama V, Matsuyama S, Sadofsky MJ. Ku70 is stabilized by increased cellular SUMO. Biochem Biophys Res Commun. 2008; 366: 263–268. 18062920 10.1016/j.bbrc.2007.11.136 PMC2212819

[31] Yurchenko V, Xue Z, Sadofsky MJ. SUMO modification of human XRCC4 regulates its localization and function in DNA double-strand break repair. Mol Cell Biol. 2006; 26: 1786–1794. 16478998 10.1128/MCB.26.5.1786-1794.2006 PMC1430232

[32] Altschul SF, Gish W, Miller W, Myers EW, Lipman DJ. Basic local alignment search tool. J Mol Biol. 1990; 215: 403–410. 2231712 10.1016/S0022-2836(05)80360-2

[33] Keeling PJ, Burki F, Wilcox HM, Allam B, Allen EE, Amaral-Zettler LA, et al The Marine Microbial Eukaryote Transcriptome Sequencing Project (MMETSP): illuminating the functional diversity of eukaryotic life in the oceans through transcriptome sequencing. PLOS Biol. 2014; 12: e1001889 10.1371/journal.pbio.1001889 24959919 PMC4068987

[34] Katoh K, Misawa K, Kuma K, Miyata T. MAFFT: a novel method for rapid multiple sequence alignment based on fast Fourier transform. Nucleic Acids Res. 2002; 30: 3059–3066. 12136088 10.1093/nar/gkf436 PMC135756

[35] Hall TA. BioEdit: a user-friendly biological sequence alignment editor and analysis program for Windows 95/98/NT. Nucl Acids Symp Ser. 1999; 41: 95–98.

[36] Stamatakis A. RAxML version 8: a tool for phylogenetic analysis and post-analysis of large phylogenies. Bioinformatics. 2014; 30: 1312–1313. 10.1093/bioinformatics/btu033 24451623 PMC3998144

[37] Lartillot N, Lepage T, Blanquart S. PhyloBayes 3: a Bayesian software package for phylogenetic reconstruction and molecular dating. Bioinformatics. 2009; 25: 2286–2288. 10.1093/bioinformatics/btp368 19535536

[38] Rice DW, Palmer JD. An exceptional horizontal gene transfer in plastids: gene replacement by a distant bacterial paralog and evidence that haptophyte and cryptophyte plastids are sisters. BMC Biol. 2006; 4: 31 16956407 10.1186/1741-7007-4-31 PMC1570145

[39] Spreitzer RJ, Salvucci ME. RuBisCO: structure, regulatory interactions, and possibilities for a better enzyme. Annu Rev Plant Biol. 2002; 53: 449–475. 12221984 10.1146/annurev.arplant.53.100301.135233

[40] Keller M, Chan RL, Tessier LH, Weil JH, Imbault P. Post-transcriptional regulation by light of the biosynthesis of *Euglena* ribulose-1,5-bisphosphate carboxylase/oxygenase small subunit. Plant Mol Biol. 1991; 17: 73–82. 1907872 10.1007/BF00036807

[41] Knight JR, Willis AE, Milner J. Active regulator of SIRT1 is required for ribosome biogenesis and function. Nucleic Acids Res. 2013; 41: 4185–4197. 10.1093/nar/gkt129 23462953 PMC3627601

[42] Doyle CM, Rumfeldt JA, Broom HR, Broom A, Stathopulos PB, Vassall KA, et al Energetics of oligomeric protein folding and association. Arch Biochem Biophys. 2013; 531: 44–64. 10.1016/j.abb.2012.12.005 23246784

[43] Vesteg M, Vacula R, Burey S, Löffelhardt W, Drahovská H, Martin W, et al Expression of nucleus-encoded genes for chloroplast proteins in the flagellate *Euglena gracilis*. J Eukaryot Microbiol. 2009; 56: 159–166. 10.1111/j.1550-7408.2008.00383.x 19457056

[44] Rikin A, Schwartzbach SD. Extremely large and slowly processed precursors to the *Euglena* light-harvesting chlorophyll a/b binding proteins of photosystem II. Proc Natl Acad Sci U S A. 1988; 85: 5117–5121. 16593956 10.1073/pnas.85.14.5117 PMC281699

[45] Muchhal US, Schwartzbach SD. Characterization of a *Euglena* gene encoding a polyprotein precursor to the light-harvesting chlorophyll a/b-binding protein of photosystem II. Plant Mol Biol. 1992; 18: 287–299. 1731990 10.1007/BF00034956

[46] Nowitzki U, Gelius-Dietrich G, Schwieger M, Henze K, Martin W. Chloroplast phosphoglycerate kinase from *Euglena gracilis*: endosymbiotic gene replacement going against the tide. Eur J Biochem. 2004; 271: 4123–4131. 15479241 10.1111/j.1432-1033.2004.04350.x

[47] Koziol AG, Durnford DG. *Euglena* light-harvesting complexes are encoded by multifarious polyprotein mRNAs that evolve in concert. Mol Biol Evol. 2008; 25: 92–100. 17947344 10.1093/molbev/msm232

[48] Zhang H, Lin S. Complex gene structure of the form II RuBisCO in the dinoflagellate *Prorocentrum minimum* (Dinophyceae). J Phycol. 2003; 39: 1160–1171.

[49] Enomoto T, Sulli C, Schwartzbach SD. A Soluble chloroplast protease processes the *Euglena* polyprotein precursor to the light harvesting chlorophyll a/b binding protein of photosystem II. Plant Cell Physiol. 1997; 38: 743–746.

[50] Song J, Tan H, Perry AJ, Akutsu T, Webb GI, Whisstock JC, et al PROSPER: an integrated feature-based tool for predicting protease substrate cleavage sites. PLOS ONE. 2012; 7: e50300 10.1371/journal.pone.0050300 23209700 PMC3510211

[51] Lin MT, Occhialini A, Andralojc PJ, Parry MA, Hanson MR. A faster RuBisCO with potential to increase photosynthesis in crops. Nature. 2014; 513: 547–550. 10.1038/nature13776 25231869 PMC4176977

[52] Kanevski I, Maliga P, Rhoades DF, Gutteridge S. Plastome engineering of ribulose-1,5-bisphosphate carboxylase/oxygenase in tobacco to form a sunflower large subunit and tobacco small subunit hybrid. Plant Physiol. 1999; 119: 133–142. 9880354 10.1104/pp.119.1.133 PMC32212

[53] Stotz M, Mueller-Cajar O, Ciniawsky S, Wendler P, Hartl FU, Bracher A, et al Structure of green-type RuBisCO activase from tobacco. Nat Struct Mol Biol. 2011; 18: 1366–1370. 10.1038/nsmb.2171 22056769

[54] Hauser T, Bhat JY, Milicic G, Wendler P, Hartl FU, Bracher A, et al Structure and mechanism of the RuBisCO-assembly chaperone Raf1. Nat Struct Mol Biol. 2015; 22: 720–728. 10.1038/nsmb.3062 26237510

[55] Feiz L, Williams-Carrier R, Belcher S, Montano M, Barkan A, Stern DB. A protein with an inactive pterin-4a-carbinolamine dehydratase domain is required for RuBisCO biogenesis in plants. Plant J. 2014; 80: 862–869. 10.1111/tpj.12686 25279696

[56] Wheatley NM, Sundberg CD, Gidaniyan SD, Cascio D, Yeates TO. Structure and identification of a pterin dehydratase-like protein as a ribulose-bisphosphate carboxylase/oxygenase (RuBisCO) assembly factor in the alpha-carboxysome. J Biol Chem. 2014; 289: 7973–7981. 10.1074/jbc.M113.531236 24459150 PMC3953307

[57] Imker HJ, Fedorov AA, Fedorov EV, Almo SC, Gerlt JA. Mechanistic diversity in the RuBisCO superfamily: the "enolase" in the methionine salvage pathway in *Geobacillus kaustophilus*. Biochemistry. 2007; 46: 4077–4089. 17352497 10.1021/bi7000483

[58] Imker HJ, Singh J, Warlick BP, Tabita FR, Gerlt JA. Mechanistic diversity in the RuBisCO superfamily: a novel isomerization reaction catalyzed by the RuBisCO-like protein from *Rhodospirillum rubrum*. Biochemistry. 2008; 47: 11171–11173. 10.1021/bi801685f 18826254 PMC2597038

[59] Machado MA, Zetsche K. A structural, functional and molecular analysis of plastids of the holoparasites *Cuscuta reflexa* and *Cuscuta europaea*. Planta. 1990; 181: 91–96. 10.1007/BF00202329 24196679

[60] Dey S, North JA, Sriram J, Evans BS, Tabita FR. *In vivo* studies in *Rhodospirillum rubrum* indicate that ribulose-1,5-bisphosphate carboxylase/oxygenase (RuBisCO) catalyzes two obligatorily required and physiologically significant reactions for distinct carbon and sulfur metabolic pathways. J Biol Chem. 2015; 290: 30658–30668. 10.1074/jbc.M115.691295 26511314 PMC4692197

[61] Price GD, Howitt SM, Harrison K, Badger MR. Analysis of a genomic DNA region from the cyanobacterium *Synechococcus* sp. strain PCC7942 involved in carboxysome assembly and function. J Bacteriol. 1993; 175: 2871–2879. 8491708 10.1128/jb.175.10.2871-2879.1993 PMC204604

