# Supplementary material for: RuBisCO in Non-Photosynthetic Alga Euglena longa: Divergent Features, Transcriptomic Analysis and Regulation of Complex Formation
Source: PLoS One. 2016 Jul 8;11(7):e0158790. doi: 10.1371/journal.pone.0158790 (PMC4938576; doi:10.1371/journal.pone.0158790)
Supplement: S1 Fig — (PDF) [file pone.0158790.s001.pdf]

A

```
N1                                                  gtctatttttttcgagccaaa    22
P1        M  R  L  D  R  Q  P  L  L  H  V  E  K  R  M  P  T  S  L  W  L  V  V  G  A  V  I
      atgcgattggaccgtcagccactgctgcatgttgagaagcgaatgccaacatccctgtggttggttgttggtgcagtgattg   104
          A  A  V  C  V  V  F  A  A  S  H  N  G  T  H  L  S  A  T  S  P  P  I  L  T  T  T
     105 ctgctgtttgtgttgtttttgctgcttcccacaatggaacacacctgtcagcaacttcaccaccaatccttacaaccacc   184
          S  T  V  R  I  P  V  V  Q  N  F  E  P  G  L  T  A  S  S  N  H  L  S  N  G  I  P
     185 tcaactgtgcggattccagtggtacagaatttgaaccaggcctgacagcctcaagcaaccacttatcaaatggaattcc   264
          P  L  G  D  S  A  G  T  E  S  A  S  R  S  F  V  A  S  A  I  L  F  P  L  C  G
     265 tcccttgggtgacagtgctgggacagagagtgcatcccgcagttttgttgcgagtgcaatcctgtttccccttgtggac   344
         L  L  A  T  V  A  F  I  M  A  K  K  N  P  Q  T  T  S  L  L  S  I  A  S  K  K  D
     345 ttcttgcaaccgtggcttttataatggcaaagaagaatccacaaacaacatctctcctctccatcgcgtccaagaaggat   424

P1           M  E  V  W  S  P  I  N  N  R  K  F  E  T  F  S  F  L  P  P  M  T  D  E  Q  I  S
PX           .  .  .  .  N  R  S  I  E  .  T  S  K  .  L  T  -  -  -  -  -  P  E  .  R  F  T
P8           .  .  .  .  .  .  .  .  .  .  .  .  .  .  .  .  .  .  .  .  .  .  .  .  .  .  .
N1       425 atggaggtgtggagccccatcaacaacaggaagttcgagaccttctcctttctgcctcccatgactgatgagcagatttc   504
N2       845 ................................................................................   924
N3      1265 ................................................................................  1344
NX         x ............a..gttc..t.g.g..a.cc.caa.a..t..ga..---------------c.c..a....g.t..a.  x+64
NX+1   x+387 ................................................................................ x+466
N8     y-401 ................................................................................ y-322

P1            K  E  V  D  M  I  I  N  K  G  Y  S  P  F  L  E  F  A  A  P  E  N  S  S  I  S
PX            R  S  L  E  R  .  V  K  E  .  L  F  .  G  V  .  Y  .  P  .  R  .  C  F  R  A
P8            .  .  .  .  .  .  .  .  .  .  .  .  .  .  .  .  .  .  .  .  .  .  .  .  .  .
N1       505 caaggaggtggacatgatcatcaacaaggggtattccccttcctggagtttgctgcccccgagaacagcagcatttcca   584
N2       925 ................................................................................  1004
N3      1345 ................................................................................  1424
NX      x+65 ..g.tc.c.t..g.ga..tg.a..gg....actc.t....ggtg.t..a.ac..cc....tcg...tt..tt.cgcg... x+129
NX+1   x+467 ................................................................................ x+546
N8     y-321 ................................................................................ y-242

P1          S  E  S  T  T  R  F  S  G  T  T  S  G  Y  Y  D  N  R  Y  W  T  M  W  K  L  P  M
PX          K  S  C  .  A  N  .  A  D  P  -  -  -  -  -  .  S  .  D  .  .  .  .  .  .  .  .
P8          .  .  .  .  .  .  .  .  .  .  .  .  .  .  .  .  .  .  .  .  .  .  .  .  .  .  .
N1       585 gtgagagcaccacccgcttctctggcaccacctctggctactacgacaaccggtactggacgatgtggaagctgcccatg   664
N2      1005 ................................................................................  1084
N3      1425 .........................g..g..t................................................  1504
NX     x+130 agagct....ag.gaa....g...a.c..---------------....g....g.......................... x+209
NX+1   x+547 ................................................................................ x+626
N8     y-241 ................................................................................ y-162

P1           F  G  C  T  D  P  S  Q  V  L  K  E  I  D  E  C  C  K  T  F  P  Q  C  Y  V  R  L
PX           .  .  .  .  .  .  N  .  .  .  .  .  V  I  .  .  .  .  I  Y  .  .  .  F  .  .  .
P8           .  .  .  .  .  .  .  .  .  .  .  .  .  .  .  .  .  .  .  .  .  .  .  .  .  .  .
N1       665 tttggctgcactgatcccagccaggtgctgaaagaaattgatgagtgctgcaagaccttccctcagtgctatgtccgcct   744
N2      1085 ................................................................................  1164
N3      1505 ........................................--------------------------------------  1548
NX     x+210 ...................a...............gg.cat............a.t..a..........t......... x+289
NX+1   x+627 ................................................................................ x+706
N8     y-161 ................................................................................  y-82

P1            A  A  F  D  S  I  K  Q  V  Q  V  I  S  F  L  V  Q  R  P  P  S  N  V  N  M  A
PX            .  .  .  .  P  .  A  .  .  .  A  .  .  .  .  .  .  .  .  D  A  -  .  S  .  .
P8            .  .  .  .  .  .  .  .  .  .  .  .  .  .  .  .  .  .  .  .  S  G  S  T  R
N1       745 ggcagccttcgactccatcaagcaggtgcaggtgatctcgttcttggtgcagcgcccccccagcaatgtgaacatggctg   824
N2      1165 ................................................................................  1244
N3           --------------------------------------------------------------------------------
NX     x+290 ............c.....gca..........ca.................a.....ggatgct---....g........ x+366
NX+1   x+707 ...........................................................c......---- x+782
N8      y-81 ..........................................................g..gc.g..cccg.t   y-2

P1           A  M  T  G  E  K  D
PX           .  .  .  .  .  .  .
P8           *  -  -  -  -  -  -
N1       825 ccatgaccggtgagaaggat    844
N2      1245 ....................   1264
N3           --------------------
NX     x+367 ....................  x+386
NX+1         --------------------
N8       y-1 ga------------------ y>4000

N8       y+1   ctgctgctgtaatttttatttcgcatcgagcttgtggttgacatttgtgcaccgcaatgtgacacaccaaaggcggac  y+78
        y+79 acacctgggaaggagtacaggtttccattcagctggtagaaacccgcattccactgcttcaccaatgctatccagtgctc y+158
       y+159 acgcagctgcattcacactcaccaagggaggataggatttgtacaacagtcaacactgcaacattcgtgccgcatctttt y+238
       y+239 ttggtgaagtgcagaagattgcccattgaacaatgcccaagtgtgttgttgtgcaaat                        y+296
```

B

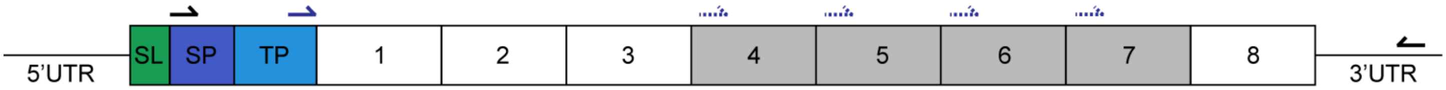

C

```
Euglena longa divergent                MEVWNRSIERTSKTLT-----PEERFTRSLERIVKEGLFPGVEYAPPRNC
Euglena longa canonical                MEVWSPINNRKFETFSFLPPMTDEQISKEVDMIINKGYSPFLEFAAPENS
Euglena gracilis                       MKVWNPVNNKKFETFSYLPPLSDAQIAKQVDMIIAKGLSPCLEFAAPENS
Eutreptiella gymnastica NIES-381       MKVWNPVNNKKFETFSYLPPLSNAEIARQVDFIIRNGLAPCLEFAPEECS
Chlamydomonas reinhardtii              MMVWTPVNNKMFETFSYLPPLTDEQIAAQVDYIVANGWIPCLEFAEADKA
Haematococcus pluvialis                MMVWQPINNKQYETFSYLPPLTSDQIARQVDYVVGNGWIPCLEFADASQA
Auxenochlorella protothecoides         MLVWTPTNNKMFETFSFLPPLSDDQISRQVDYIISNKWIPCLEFIDDPFP

Euglena longa divergent                FRAKSCTANFADP-----DSRDWTMWKLPMFGCTDPNQVLKEVIECCKIY
Euglena longa canonical                SISSESTTRFSGTTSGYYDNRYWTMWKLPMFGCTDPSQVLKEIDECCKTF
Euglena gracilis                       FIANDNTVRFSGTAAGYYDNRYWTMWKLPMFGCTDASQVLREISECRRAY
Eutreptiella gymnastica NIES-381       FVDDVATVRFQGSTAGYYDNRYWTMWKLPMFGCSSAQEVLREVQECTRHY
Chlamydomonas reinhardtii              YVSNESAIRFGSVSCLYYDNRYWTMWKLPMFGCRDPMQVLREIVACTKAF
Haematococcus pluvialis                YVSNASTVRFGSVSACYYDNRYWTLWKLPMFGCTDPSAVLTEISRASKAF
Auxenochlorella protothecoides         YVAQENTIRMGAVASNYYDNRYWTMWKLPMFGCNDGSQVLTEVGNCRKAF

Euglena longa divergent                PQCFVRLAAFDPIAQVQAISFLVQRP----DA-VSMAAMTGEKD
Euglena longa canonical                PQCYVRLAAFDSIKQVQVISFLVQRP----PSNVNMAAMTGEKD
Euglena gracilis                       PQCYVRLAAFDSVKQVQVISFVVQRPSGSSSSSWGMAAMTGEKD
Eutreptiella gymnastica NIES-381       PGCYVRLAAFDSIKQVQVVSFIVHQP----GQ-TAAWATTGEKD
Chlamydomonas reinhardtii              PDAYVRLVAFDNQKQVQIMGFLVQRP------------------
Haematococcus pluvialis                PQAYIRMVAFDNVRQVQIMSFLVQRP------------------
Auxenochlorella protothecoides         PDAYIRLVAFDNVRQVQIGGFLVHRP------------------
```

Figure S1. RuBisCO small subunit in *E. longa*. 1A**.** The partially reconstructed sequence of the *RbcS* mRNA and the corresponding RBCS precursor polyprotein in *Euglena longa*. The mRNA starts with a spliced leader sequence (in green), as is typical for mRNA molecules in Euglenozoa. Downstream is the rest of the 5'-UTR followed by the first part of the sequence encoding a predicted tripartite plastid-targeting sequence (amino acid residues in blue: dark blue represents a signal and light blue a transit peptide). The middle part of the figure (shaded in darker grey) shows an alignment of determined complete or partial sequences of repeats encoding the RBCS monomer (amino acid residues on top in black) followed by the linker decapeptide (amino acid residues on top in red). Sequences of the first two repeats (N1 and N2) are complete, the third repeat (N3) lacks the 3'-end of the sequence. NX (nucleotide sequence) and PX (protein sequence) correspond to the divergent repeat that is a part of the molecule, but whose exact position with respect to other repeats could not be determined. It is followed by a canonical repeat (NX+1; note that only a part of the linker sequence following this repeat was determined). The terminal repeat is denoted N8, assuming that the total number of repeats is eight (see the text). This last repeat terminates with a stop codon (TGA, in bold and italics) and is followed by a long 3'-UTR. Only the sequence of the first repeat is shown in full, in the other repeats dots represent the same nucleotides (amino acid residues) as in the first repeat, letters indicate substitutions as compared to the first repeat, dashes in black represent deletions, dashes in gray correspond to missing data. Position coordinates are indicated on the left and on the right of each line. The actual position of the beginning of the divergent repeat (NX) is unknown, so it is indicated as "x" and the positions downstream of it are counted accordingly. Likewise, the exact coordinates of the 3'-region of the mRNA molecule are unknown, so the last nucleotide of the stop codon was arbitrarily indicated as "y" and positions upstream and downstream are counted accordingly. Regions corresponding to primers used for PCR amplification are indicated by black boxes, regions corresponding to internal primers used for sequencing are indicated by dark blue boxes. 1B. Schematic representation of the *RbcS* transcript. SL, splice leader sequence; SP, signal peptide sequence; TP, transit peptide sequence. The grey box represents a divergent subunit, although its exact position was not determined. Arrows indicate primers used for PCR and sequencing as in 1A. The dashed arrow represents a primer that anneals to the divergent subunit. 1C. Alignment of RBCS sequences from euglenophytes and selected green algae. Only the region corresponding to the mature processed RBCS protein is shown, for euglenophytes also the linker decapeptide is included at the end of the sequence. Both the canonical and the divergent forms of RBCS are shown for *E. longa*.
