# Supplementary material for: RuBisCO in Non-Photosynthetic Alga Euglena longa: Divergent Features, Transcriptomic Analysis and Regulation of Complex Formation
Source: PLoS One. 2016 Jul 8;11(7):e0158790. doi: 10.1371/journal.pone.0158790 (PMC4938576; doi:10.1371/journal.pone.0158790)
Supplement: S2 Fig — (PDF) [file pone.0158790.s002.pdf]

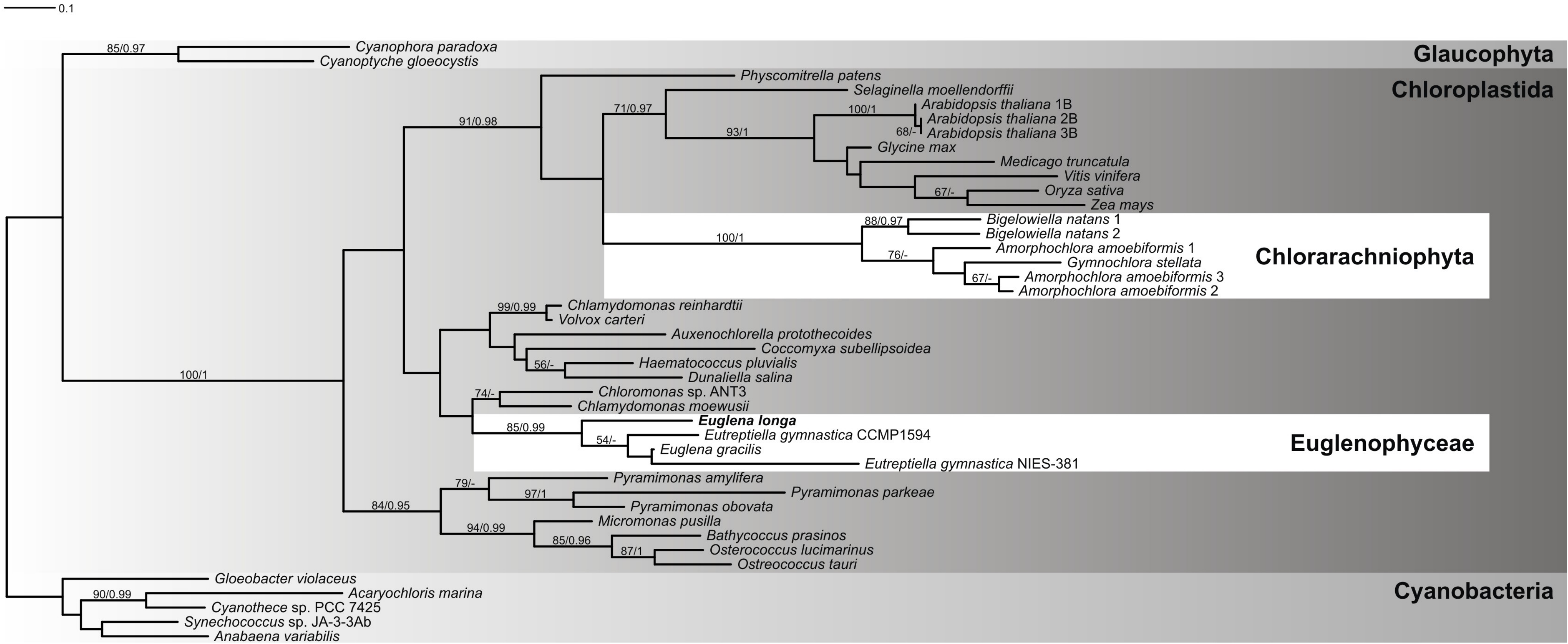

Figure S2. Phylogenetic tree of RBCS protein sequences. The maximum-likelihood tree was inferred with RAxML using the LG+Γ substitution model. The bootstrap support values and posterior probabilities (from PhyloBayes) are indicated at branches when higher than 50% and 0.95, respectively. Clades with organisms possessing secondary plastids are boxed. *E. longa* is in bold.
