# Supplementary material for: RuBisCO in Non-Photosynthetic Alga Euglena longa: Divergent Features, Transcriptomic Analysis and Regulation of Complex Formation
Source: PLoS One. 2016 Jul 8;11(7):e0158790. doi: 10.1371/journal.pone.0158790 (PMC4938576; doi:10.1371/journal.pone.0158790)
Supplement: S4 Fig — (PDF) [file pone.0158790.s004.pdf]

```
>E.longa_GroEL
MLAANSRKIWSWGLVASALAILAVGALSATPADFTELLANTQMSKPIQMPAVA
NSPSRMGSLHRAMPYTVAGSPKLDELYATASTPVKSRGPWVPIAFTALVSSVALMVAAFH
QRRKKAVAVAAIAGSKWNFPMYSAIQPPISRRTTTTCHAAKEIIFNETGEALKRMQAGVD
KLSSCVGVTLGPKGRNVVLESNYGAPKIVNDGVTIAREIDLQDPVEAIGAKLVRQASQKT
NDQAGDGTTTAIILSHAFITEGMKIVAAGANPVQLTRGMEKTVAHLVQELKKLSKEVEDN
ELAYVASVSAGGNIEVGEMISKAMARVGRRGVITMEESRGVDNDMYVVEGMQFERGFMSP
YFVTDAERNLVHYDNARLLLVDMKITSARDVVNVLELSLQEGFPLIILAEGIEQEALATL
VVNRLRGSLKVCAIKAPGFGERKTQYLEDIAILTGGTVVKAELGLQLSKVGKEVLGTAAR
VEITKDSTTIVGDGSTAPEVQARIRQIEGLLEAATADYEKDRLRERIARLSGGVAIIQVG
ANTETELKDKKLRVEDALMATKAAVEEGIVIGGGCTLLALADRVDDIKGKLENDEQKVGA
EIIKRALSYPLRLVAHNAGENGFVVLENVRKLGKDMGFNAATGQYEDLMKSGIIDPTKVI
RCCLENACSVAKLFLTSEAVVVEIPEKGAGAQKPNPADSSFTL-

>E.longa_GroES
MSPVLYGAVGRPAIPPQPRSALPLLAAGALCAGALLGVVLSSADPTSLFTSPS
LTAPRHATLAVSRAHRGHGPFTEPIRTVPDTTANAVEALHAEVSAQDETADQALPSTATA
FWWALLAGCAGAMLSAMAGLFAKPQRPKSSSVHAVPLAMVAASGAHDYAGDLKKLTPLRD
LVLVRVAPMEGTTTGGLVLPDVMQTKPTSGDVVAVGPKVSELKGEECILYSKFGVNLTEL
EVGGQAHVLIREADIIGVLPRSPASSKDTPELKPLLDKVLVRKDAEPTNKLSGLILQAAD
ADAKYITGEVVAVGGGWTDSNGQFHALTVLPGQKVLFSKYAGDKLPTPGGPEYVCVAESE
ILGVL-
```

Figure S4. GroEL/GroES homologs in *E. longa*. Protein sequences of GroEL/GroES homologs found in the *E. longa* transcriptome.
