# Supplementary material for: RuBisCO in Non-Photosynthetic Alga Euglena longa: Divergent Features, Transcriptomic Analysis and Regulation of Complex Formation
Source: PLoS One. 2016 Jul 8;11(7):e0158790. doi: 10.1371/journal.pone.0158790 (PMC4938576; doi:10.1371/journal.pone.0158790)
Supplement: S1 Table — (PDF) [file pone.0158790.s005.pdf]

TABLE S1. List of PCR and qPCR primers used in this work. Coordinates for sequences in Fig. S1 are indicated.

NAME	SEQUENCE (5' - 3')	COORDINATES IN FIG. S1
rbcL_F_qPCR	GAAGTWAAAGGACATTATTTAAATG	-
rbcL_R_qPCR	CCACCAGACATACGAAGTGT	-
RbcS_F_qPCR	GGCTACTACGACAACCGGTACTGG	-
RbcS_R_qPCR	AACGAGATCACCTGCACCTGCTTG	-
Rrn18S_F_qPCR	CTTAGATCGCTGCCAGATCC	-
Rrn18S_R_qPCR	GGCTGTGGATTTCTCGTTGT	-
RbcS_F1	ATGCGATTGGACCGTCAGCCACTGCT	23 - 48
RbcS_F2	CTCCTCTCCATCGCGTCCAAGAAGG	397 - 422
RbcS_R	CTTCTGCACTTCACCAAAAAAGATGC	y+255 - y+230
RbcS-X_F	GGAACCGTTCCATCGAGAGAACC	x+11 - x+32
