# Supplementary material for: RuBisCO in Non-Photosynthetic Alga Euglena longa: Divergent Features, Transcriptomic Analysis and Regulation of Complex Formation
Source: PLoS One. 2016 Jul 8;11(7):e0158790. doi: 10.1371/journal.pone.0158790 (PMC4938576; doi:10.1371/journal.pone.0158790)
Supplement: S2 Table — (PDF) [file pone.0158790.s006.pdf]

TABLE S2. List of the RBCL sequences used in phylogenetic analysis. Accession numbers for GenBank entries and contig ids for MMETSP* are listed.

	ORGANISM	SOURCE (Database)	ACCESSION NUMBER/CONTIG
Euglenozoa	*Euglena longa*	GenBank	NP_074963
	Euglena gracilis Z	GenBank	NP_041936
	Euglena viridis NJ001	GenBank	YP_007517056
	Euglenaformis proxima	GenBank	YP_009032761
	Eutreptiella gymnastica K-0333	GenBank	YP_006234185
	Eutreptiella gymnastica CCMP1594	MMETSP	CAMNT_0046485463
	Eutreptia viridis	GenBank	AEQ94204
	Colacium vesiculosum	GenBank	AEW12959
	Monomorphina aenigmatica	GenBank	YP_007317188
	Strombomonas acuminata	GenBank	AEW12993
	Cryptoglena skujai	GenBank	AKL39014
	Euglena gracilis bacillaris	GenBank	AKL82391
	Euglena viridis SAG 224-17d	GenBank	AKL79017
	Euglenaria anabaena	GenBank	AKJ83339
	Monomorphina parapyrum	GenBank	AKL78929
	Trachelomonas volvocina	GenBank	AKL82413
Chloroplastida	*Chlamydomonas reinhardtii*	GenBank	NP_958405
	Chlamydomonas moewusii	GenBank	ABU88322
	Arabidopsis thaliana	GenBank	NP_051067
	Oryza sativa	GenBank	YP_654221
	Ostreococcus tauri	GenBank	YP_717262
	Glycine max	GenBank	YP_538747
	Medicago truncatula	GenBank	YP_001381744
	Micromonas sp. RCC299	GenBank	ACO55540
	Zea mays	GenBank	NP_043033
	Vitis vinifera	GenBank	YP_567084
	Selaginella moellendorffii	GenBank	ADH10411
	Volvox carteri	GenBank	ACY06055
	Physcomitrella patens	GenBank	NP_904194
	Pyramimonas amylifera	MMETSP	CAMNT_0041901623
	Pyramimonas obovata	MMETSP	CAMNT_0006873903
	Pyramimonas parkeae	GenBank	YP_002600925
	Dunaliella salina	GenBank	ACS95083
	Haematococcus pluvialis	GenBank	ACJ64122
	Chloromonas sp. ANT3	GenBank	AAD00447
	Chlorella vulgaris	GenBank	NP_045897
	Auxenochlorella protothecoides	GenBank	YP_009019341
	Aneura mirabilis	GenBank	YP_001687224
	Orobanche corymbosa	GenBank	AAC49722
	Orobanche fasciculata	GenBank	AAC49723
	Orobanche californica	GenBank	YP_009108392
	Harveya capensis	GenBank	AAY98036
	Harveya stenosiphon	GenBank	AAY98035
	Harveya coccinea	GenBank	AAY98034
Glaucophyta	*Cyanophora paradoxa*	GenBank	NP_043240
	Cyanoptyche gloeocystis	MMETSP	CAMNT_0041991067
Cyanobacteria	*Synechococcus* sp. JA-3-3Ab	GenBank	Q2JV67
	Gloeobacter violaceus	GenBank	NP_925102
	Anabaena variabilis	GenBank	ABA23512
	Acaryochloris marina	GenBank	B0CCC1
	Cyanothece sp. PCC 7425	GenBank	B8HQS5
Chlorarachniophyta	*Bigelowiella natans*	GenBank	YP_778601
	Lotharella sp. CCMP622	GenBank	AGY61412

*MMETSP - Marine Microbial Eukaryote Transcriptome Sequencing Project (http://marinemicroeukaryotes.org/)
