# Supplementary material for: RuBisCO in Non-Photosynthetic Alga Euglena longa: Divergent Features, Transcriptomic Analysis and Regulation of Complex Formation
Source: PLoS One. 2016 Jul 8;11(7):e0158790. doi: 10.1371/journal.pone.0158790 (PMC4938576; doi:10.1371/journal.pone.0158790)
Supplement: S3 Table — (PDF) [file pone.0158790.s007.pdf]

**TABLE S3.** List of the RBCS sequences used in phylogenetic analysis. Accession numbers for GenBank entries and contig ids for MMETSP* are listed.

| | ORGANISM | SOURCE (Database) | ACCESSION NUMBER/CONTIG |
|---|---|---|---|
| Euglenozoa | *Euglena longa* | GenBank | KT818574 |
| | *Euglena gracilis* | GenBank | 132143 |
| | *Eutreptiella gymnastica* CCMP1594 | MMETSP | CAMNT_0046443149 |
| | *Eutreptiella gymnastica* NIES-381 | MMETSP | CAMNT_0000678951 |
| Chloroplastida | *Chlamydomonas reinhardtii* | GenBank | XP_001702409 |
| | *Arabidopsis thaliana* 1B | GenBank | NP_198659 |
| | *Arabidopsis thaliana* 2B | GenBank | NP_198658 |
| | *Arabidopsis thaliana* 3B | GenBank | NP_001119331 |
| | *Oryza sativa* | GenBank | AAR19268 |
| | *Ostreococcus tauri* | GenBank | XP_003084235 |
| | *Osterococcus lucimarinus* | GenBank | XP_001422165 |
| | *Glycine max* | GenBank | AF303939 |
| | *Medicago truncatula* | GenBank | KEH21539 |
| | *Micromonas pusilla* | GenBank | XP_003064624 |
| | *Zea mays* | GenBank | CAA70416 |
| | *Vitis vinifera* | GenBank | XP_002276967 |
| | *Selaginella moellendorffii* | GenBank | XP_002991566 |
| | *Volvox carteri* | GenBank | XP_002955679 |
| | *Physcomitrella patens* | GenBank | BAC87878 |
| | *Pyramimonas amylifera* | MMETSP | CAMNT_0041894263 |
| | *Pyramimonas obovata* | MMETSP | CAMNT_0006872053 |
| | *Pyramimonas parkeae* | MMETSP | CAMNT_0035469105 |
| | *Chlamydomonas moewusii* | GenBank | P17537 |
| | *Dunaliella salina* | GenBank | AAU93597 |
| | *Haematococcus pluvialis* | GenBank | ABB17556 |
| | *Chloromonas* sp. | GenBank | AAD00448 |
| | *Bathycoccus prasinos* | GenBank | XP_007511178 |
| | *Coccomyxa subellipsoidea* | GenBank | XP_005648578 |
| | *Auxenochlorella protothecoides* | GenBank | KFM22871 |
| Glaucophyta | *Cyanophora paradoxa* | GenBank | NP_043239 |
| | *Cyanoptyche gloeocystis* | MMETSP | CAMNT_0041991067 |
| Cyanobacteria | *Synechococcus* sp. JA-3-3Ab | GenBank | YP_474645 |
| | *Gloeobacter violaceus* | GenBank | NP_925104 |
| | *Anabaena variabilis* | GenBank | YP_324405 |
| | *Acaryochloris marina* | GenBank | YP_001516118 |
| | *Cyanothece* sp. PCC 7425 | GenBank | WP_012628805 |
| Chlorarachniophyta | *Amorphochlora amoebiformis 1* | GenBank | ABW97363 |
| | *Amorphochlora amoebiformis 2* | GenBank | ABW97364 |
| | *Amorphochlora amoebiformis 3* | GenBank | ABW97365 |
| | *Bigelowiella natans 1* | GenBank | AAP79188 |
| | *Bigelowiella natans 2* | GenBank | AAP79189 |
| | *Gymnochlora stellata* | GenBank | ACF24549 |

*MMETSP - Marine Microbial Eukaryote Transcriptome Sequencing Project (http://marinemicroeukaryotes.org/)
