# Supplementary material for: RuBisCO in Non-Photosynthetic Alga Euglena longa: Divergent Features, Transcriptomic Analysis and Regulation of Complex Formation
Source: PLoS One. 2016 Jul 8;11(7):e0158790. doi: 10.1371/journal.pone.0158790 (PMC4938576; doi:10.1371/journal.pone.0158790)
Supplement: S4 Table — (PDF) [file pone.0158790.s008.pdf]

TABLE S4. List of the RCA sequences used in phylogenetic analysis. Accession numbers for GenBank entries, contig ids for MMETSP[a], and EST id for the CGP[b] are listed.

	ORGANISM	SOURCE (Database)	ACCESSION NUMBER/CONTIG
Euglenozoa	*Euglena longa*	GenBank	KT818573
	Euglena gracilis	GenBank	EC682479; EC679240; EG565129
	Eutreptiella gymnastica CCMP1594	MMETSP	CAMNT_0046458117
	Eutreptiella gymnastica NIES-381	MMETSP	CAMNT_0000686649
Chloroplastida	*Chlamydomonas reinhardtii*	GenBank	XP_001692244
	Arabidopsis thaliana	GenBank	NP_565913
	Oryza sativa	GenBank	P93431
	Ostreococcus tauri	GenBank	XP_003078787
	Osterococcus lucimarinus	GenBank	ABO95802
	Glycine max	GenBank	ADD60244
	Medicago truncatula	GenBank	XP_003604901
	Micromonas sp. RCC299	GenBank	XP_002501510
	Zea mays	GenBank	NP_001104921
	Vitis vinifera	GenBank	XP_002270571
	Volvox carteri	GenBank	XP_002951971
	Physcomitrella patens	GenBank	EDQ82463
	Pycnococcus provasolii	MMETSP	CAMNT_0053510249
	Pyramimonas amylifera	MMETSP	CAMNT_0041893585
	Pyramimonas obovata	MMETSP	CAMNT_0006861995
	Pyramimonas parkeae	MMETSP	CAMNT_0035452761
	Coccomyxa subellipsoidea	GenBank	XP_005651127
	Bathycoccus prasinos	GenBank	XP_007512493
Glaucophyta	*Cyanophora paradoxa*	CGP	EST111910
	Cyanoptyche gloeocystis	MMETSP	CAMNT_0041989161
	Gloeochaete witrockiana	MMETSP	CAMNT_0038655913
Cyanobacteria	*Synechococcus* sp. JA-3-3Ab	GenBank	WP_011429335
	Anabaena sp. CA	GenBank	Q06721
	Nostoc sp. PCC 7120	GenBank	P58555
	Arthrospira sp. PCC 8005	GenBank	CDM97162
	Kamptonema sp.	GenBank	WP_007357073

[a]MMETSP - Marine Microbial Eukaryote Transcriptome Sequencing Project (http://marinemicroeukaryotes.org/)

[b]CGP - Cyanophora Genome Project (http://cyanophora.rutgers.edu/cyanophora/home.php)
